# Color polymorphism and mating trends in a population of the alpine leaf beetle *Oreina gloriosa*

**DOI:** 10.1371/journal.pone.0298330

**Published:** 2024-03-26

**Authors:** Angela Roggero, Daniele Alù, Alex Laini, Antonio Rolando, Claudia Palestrini

**Affiliations:** 1 Department of Life Sciences and Systems Biology, University of Turin, Torino, Italy; 2 NBFC, National Biodiversity Future Center, Palermo, Italy; University of Saskatchewan College of Agriculture and Bioresources, CANADA

## Abstract

The bright colors of Alpine leaf beetles (Coleoptera, Chrysomelidae) are thought to act as aposematic signals against predation. Within the European Alps, at least six species display a basal color of either blue or green, likely configuring a classic case of müllerian mimicry. In this context, intra-population color polymorphism is paradoxical as the existence of numerous color morphs might hamper the establishment of a search image in visual predators. Assortative mating may be one of the main factors contributing to the maintenance of polymorphic populations. Due to the marked iridescence of these leaf beetles, the perceived color may change as the viewing or illumination angle changes. The present study, conducted over three years, involved intensive sampling of a population of *Oreina gloriosa* from the Italian Alps and applied colorimetry and a decision tree method to identify the color morphs in an objective manner. The tertiary sex ratio of the population was biased in favor of males, suggesting that viviparous females hide to give birth. Seven color morphs were identified, and their frequencies varied significantly over the course of the study. Three different analyses of mating (JMating, QInfomating, and Montecarlo simulations) recognized a general trend for random mating which coexists with some instances of positive and negative assortative mating. This could help explain the pre-eminence of one morph (which would be favored because of positive selection due to positive assortative mating) in parallel with the persistence of six other morphs (maintained due to negative assortative mating).

## Introduction

Color polymorphism can be defined as the existence of two or more distinct, genetically controlled, color morphs within populations that are not due to chance events [1]. This widespread phenomenon has long puzzled ecologists and entomologists, who have focused on the role of predator-prey interactions to explain the maintenance of different color morphs [2]. Recent research has even suggested that prey color polymorphism and iridescence may be adaptive. The existence of grasshopper morphs with different degrees of camouflage may help these insects defend themselves against predation at both the individual and population level [3]. Iridescence has also been suggested to act as camouflage in jewel beetles [4].

Leaf beetles (Coleoptera, Chrysomelidae) are widespread, often colorful and iridescent insects which may present color polymorphism. However, these species do not employ color as camouflage for hiding from predators. On the contrary, their bright metallic colors are thought to constitute aposematic signals that warn predators of the leaf beetle’s chemical defenses stored in the body and the specialized elytral exocrine glands of adult specimens [5]. The leaf beetle’s defensive chemistry may depend on the choice of host plants. Within the genus *Oreina* Chevrolat, 1837, for instance, certain species are defended by pyrrolizidine alkaloids (PAs) acquired by eating the leaves of Asteraceae and/or Apiaceae, while others defend themselves by synthesizing *de novo* cardenolides (autogenous defense) [6–10]. These chemicals render any caught individual so toxic and distasteful to the predator that it is triggered, in theory, to spit it out immediately. Indeed, evidence supporting aposematism has been provided by several experimental tests carried out in both natural and laboratory conditions. Using tethered beetles of the species *Oreina gloriosa* (Fabricius, 1781) exposed to natural predators, it was found that the predominant color morph was associated with higher survival rates [11], supporting the hypothesis that predators can recognize the most abundant local color morph. By modulating laboratory conditions, it was showed that specular reflectance and glossiness could amplify the warning signal of *Oreina cacaliae* (Schrank, 1785), augmenting avoidance learning in insectivorous birds [12]. Differential bird responses to color morphs may affect morph frequencies in *Chrysomela lapponica* Linnaeus, 1758 [13]. Finally, the color aposematism of some leaf beetle species of the genus *Oreina* was recently framed into the broader context of müllerian mimicry. The species of this largely Alpine, Palearctic genus [14] are colorful and chemically defended [8]. Within the Alps, at least six species of *Oreina* display a basal color of either blue or green and they may coexist in patchily distributed locations [12, 15, 16]. Laboratory assays of predatory behavior suggested that bird predators learned to associate color with chemical defenses, and that learned avoidance of the green morph of one species protected green morphs of another species, configuring a classic case of müllerian mimicry [16]. All the abovementioned studies sustain the possibility that color morph frequencies are controlled by predators. However, the maintenance of polymorphism cannot depend on predator-prey relationships since the existence of two or numerous color morphs within one population would hamper the establishment of a search image in a visual predator [17]. Moreover, aposematic signals work best when easily detectable and memorable [18]. The within-population polymorphism seen in many leaf beetle species appears to be paradoxical in the context of warning color and mimicry [11]. Instead, the maintenance of color polymorphism in leaf beetles may depend on several distinct factors, such as population dynamics [19], climatic conditions, and thermal effects [20], as well as a plethora of sexual and natural selection pressures [11, 21].

Mating may also affect the maintenance of multiple color morphs. Vertebrates and invertebrates often mate with those resembling themselves, a phenomenon described as positive assortative mating, while negative mating may be rare [22, 23]. Populations engaging in assortative mating may be more resilient and productive than those mating randomly [24]. The relationship between mating and polymorphism maintenance can be analyzed by considering the effect of mating on frequency-dependent natural and sexual selection. Intrapopulation polymorphism can only be maintained by negative frequency-dependent selection, in which selection favors the less frequent variant; on the other hand, positive frequency-dependent selection inevitably causes the fixation of the most frequent variant [11, 25, 26]. Positive assortative mating can cause positive frequency-dependent sexual selection, while negative assortative mating can cause negative frequency-dependent sexual selection [27–29]. Therefore, only negative assortative mating (by causing negative frequency-dependent sexual selection) can maintain a polymorphism forever, whereas positive assortative mating (by producing a positive frequency-dependent sexual selection) will favor the most frequent color, thereby erasing any polymorphism. More sophisticated options could also exist, for example, in a heterogeneous environment with different colors adapted to different habitats, positive assortative mating could help to maintain homozygosity for color in each habitat and, at the same time, a polymorphism along the heterogeneous environment.

Positive assortative mating in leaf beetles has previously been assessed in relation to beetle size [30] and parasite load [31]. The cuticular hydrocarbon (CHC) profile of leaf beetles, which depends on host plant species, is also known to mediate mate recognition; for instance, mustard leaf beetles *Phaedon cochleariae* (Fabricius, 1792) prefer mates with a similar CHC pattern to themselves, and, therefore, individuals that feed on the same plant species [32].

In addition to the interspecific context, where leaf beetle color is used as a visual cue in predation, leaf beetle color may also play a role in the intraspecific context by influencing mate choice. For instance, when using 3D-printed models of the flea beetle *Altica fragariae* (Nakane, 1955), it was shown that color, in addition to CHCs and shape, was an important factor in female mate selection [33]. It is also plausible that the positive assortative mating of achromist (uncolored) individuals of the potato beetle *Leptinotarsa decemlineata* Say, 1824 was based on the ability to distinguish different degrees of integument melanization [34].

In leaf beetles, pigmentation is produced by epicuticular microstructures in the elytron; more specifically, it is the differences in the periodicity of reflecting layers that create the different color morphs [35–37].

Intra-population color morphs are often recognized because of their basal colors, e.g., green, or blue [11] and melanic and non-melanic forms [38]. Some attempt to identify even the rarest morphs has been made by comparing beetles with the standard color scale for gems of the Gemological Institute of America GIA [39]. One of the major problems associated with the identification of color morphs lies in the fact that, due to their marked iridescence, color may change as the angle of view or the angle of illumination changes. Therefore, color classification by sight is subject to error. Moreover, it is important to note that, to the best of our knowledge, the color morphs of these beetles have never been objectively analyzed and classified by means of instrumental color assessment.

The alpine leaf beetle *Oreina gloriosa* is mostly found in the Alps of France, Italy, Germany, Switzerland, and Austria [40]. This species is monophagous, given that both larvae and adults feed on *Imperatoria ostruthium* (Linneus, 1753), commonly known as masterwort (Apiaceae) [41–43]. Adults, which measure 8–10 mm in length, are iridescent and appear in the wild as either a bright metallic green or metallic blue color. We focused on a large population located in the northwestern Italian Alps and sampled it extensively over a period spanning three years. The main aims of the research were threefold: i) to identify the color morphs by applying an objective, instrument-assisted procedure, ii) to verify whether the sex ratio and the proportion of morphs remained stable over time, and iii) to investigate the hypothesis that these morphs pair in an assortative (negative or positive) manner. The study of mating trends is not free from experimental difficulties. One of the potential problems of any study on mating trends is the scale-of-choice effect (from here on SCE) [44–46]. The SCE exists when two assumptions are met: i) the scale of mate choice is much smaller than the sampling scale, and ii) variations in the spatial distribution of the trait considered may exist within the sampled area. Since *O*. *gloriosa* is a winged insect, capable of moving rapidly across the entire study area, we assumed the scale of mate choice to be approximatively comparable with the sampling scale. Furthermore, the presence of the two main color morphs assessed by sight (blue and green) seemed to be present in an apparently uniform way throughout the entire area. So, in the programming phase of the experimental design we assumed SCE to be negligible.

## Material and methods

### Fieldwork

The collection site (45° 49’ 25’’ N, 7° 33’ 55” E) is in the municipality of Torgnon (Valtournenche, Aosta Valley), in the northwestern Italian Alps. The adult leaf beetles sampled were inhabiting an area of larch forest (*Larix decidua* Miller, 1768) at an altitude of approx. 1900 m a.s.l. The undergrowth was primarily composed of rhododendron *Rhododendron ferrugineum* Linnaeus, 1753 and green alder *Alnus viridis* (Chaix, 1805). The host plant on which most beetles were settled, and whose leaves they were eating, was the masterwort, *I*. *ostruthium*. The leaf beetles were found as either single individuals or mating pairs, and they were extensively collected during the month of July in the years 2020, 2021, and 2022. The total area of forest sampled was approximately 800 x 100 m. This area was divided into four parts, each measuring 200 x 100 m, and each part was searched on a separate day (for a total of four collection sessions per year). During each collection session, we scanned the surface of the vegetation and collected all individuals spotted. These leaf beetles tend to stay on the visible surfaces of the broad leaves of their host plants independent of beetle color, sex, or reproduction status; therefore, our methods are unlikely biased to collecting a specific morph, sex, or occurrence modality (single vs paired). The first year was dedicated to the study of color patterns, whereas the second and third years were dedicated to the study of mating patterns, which were assessed by three different methods. In 2020, we collected all individuals without distinguishing between single and paired beetles. In 2021, we only collected mating pairs, and we applied both JMating software [47], dedicated to the analysis of sexual selection and sexual isolation effects using mating frequency data, and QInfomating software [48], which tests for sexual selection and assortative mating by calculating the best fit model and using multi-model inference techniques to estimate the values of the parameters. In 2022, we collected both mating pairs and single individuals (to calculate simulated frequencies of mating pairs) and used the data on their frequencies in Montecarlo simulations.

We collected mating pairs regardless of whether they were in the act of copulating or not. Once collected, we preserved the individuals in absolute alcohol and transported them to the laboratory where, following species identity confirmation [14, 15, 49, 50] they were sexed and color-classed using an instrument-assisted color evaluation process (see below).

### Identification of color morphs

#### Preface

As a necessary preface, it should be said that due to the accentuated iridescence of *O*. *gloriosa*, the visual perception of color varies greatly depending on the environmental context. In large assemblages in the wild, two prominent morphs have been detected, namely green and blue [11]. However, this dualism is lost when the individuals of a mating pair are compared. Here, the attribution of color becomes a question of individual perception, with different people seeing different colors, especially when it comes to specimens which may appear to be simultaneously both blue and green and in those showing an additional teal, goldish or copper tones. Indeed, in preliminary trials, the classifications proposed by the co-authors were never found to coincide. For this reason, it was absolutely necessary to develop a methodology able to identify and estimate color objectively.

#### Color rendering

The colorimeter NR60CP (3nh, Shenzhan) detected the color of each individual according to the human visible light spectrum (between 380 and 750 nm), and the software CQCS3 acquired and stored the data. The colorimeter illuminant was set at D65 (daylight at noon), with the 4 mm measuring aperture (= measurement caliper). We selected SCI (specular reflectance included) as the method of color measurement.

We conducted the color analysis within the CIE L*a*b* color space, a model for color measurement defined by the International Commission on Illumination (ICC) [51, 52], whereby the chromaticity index was defined by five variables: L* = lightness, a* = red/green coordinate, b* = yellow/blue coordinate, C* = chroma, and h* = hue angle [53]. This color space is tridimensional, thence to represent a color three parameters must be used together. Either L*a*b* or L*C*h* can be alternatively selected, since the underlaying color model is the same for both.

We measured the color of each individual three times to test the reliability of the procedure, having taken care to place the measurement caliper always on the same portion of the elytral curved surface (S1 Fig) to obtain comparable reflectance values. We calculated the Euclidean distances (ΔE*) between the three measurements using the CIE L*a*b* values. Due to the noticeable iridescence of this species [54–56], the acceptable threshold value of the difference between the measurements was set at <3.0.

Once the consistency of the repeated measurements was established, we calculated the ΔE* from each measurement *vs* an empty measurement (i.e., the standard “null”, with all the values defined equal to 0) for both the L*a*b* and the L*C*h* set of values. The mean value of the three Euclidean distances (ΔE_1_, ΔE_2_ ed ΔE_3_) provided us with an objective measurement of color for each specimen.

#### Identification and classification of color morphs

The decision tree method is known as one of the most effective ways of creating a classification system. It is user-friendly and its applicability has been verified in numerous contexts [57]. Several algorithms have been developed for classification purposes, such as the J48 classifier, one of the most widely used algorithms that implements a top-down decision tree classification (DTC) system to obtain the final output [58].

To identify leaf beetle color morphs, we applied the ΔE* L*C*h* values in a DTC using the J48 algorithm in Weka v3.8.5 software [59–61] set to the default settings. The classification was set *a priori* to increase the number of color morphs from three (i.e., the initial color classification done at sight by the same observer, DA) to seven. In that way we fitted the distribution of individuals shown in the plot of the two datasets of the ΔE* values (S2 Fig) and reduced the classification errors to negligible.

### Mating assessment

Mating assessment (random, positive, and negative assortative mating) was conducted applying: i) JMating v1.0.8 [47] and QInfomating v0.4 [48, 62], using the dataset of heterosexual mating pairs collected in 2021 and ii) Montecarlo simulations, using the dataset of heterosexual mating pairs and single individuals collected in 2022.

#### JMating

To evaluate different choice designs the mating frequency data were analyzed and a non-parametric G test applied to evaluate whether effects of sexual isolation and sexual selection were significant (separately or as a combined effect). The total Ipsi index (range between -1 and +1, with 0 = random mating) was used as the better estimator of assortative mating in the bootstrap (N_reps_ = 10, each with 100,000 iterations) resampling distribution paired with two-tail probability validation [46]. We estimated pairwise PSI coefficients (sexual isolation, defined for every pair combination as the number of observed pair types divided by the number of expected pair types from mates) [44].

#### QInfomating

To test for the presence of non-random mating in the dataset, we examined the mutual mating propensity of males and females [48], sorting them according to the seven color morphs, and evaluating the deviation from random mating. The variance of each model was multiplied by the overdispersion factor. We tested different mutual mating propensity models (random mating, mate competition and/or mate choice), using the Akaike information criterion (AICc) to identify the best-fitting model [48].

#### Montecarlo simulations

First, we calculated the frequencies of the different color morphs of each sex considering the single individuals collected in 2022. Next, by applying the observed frequencies in Montecarlo simulation, we tested for non-random mating according to color morph using a parametric bootstrap approach [63] For each sex, the expected color morphs of 437 individuals (being the number of couples found in 2022) were randomly generated using the function sample of the R software for statistical computing [64]. During the simulation, the probability of obtaining a morph was set to the observed frequency. The results for each sex were merged and the number of couples for each combination of morphs calculated. This procedure was repeated 1000 times, after which two-tailed probabilities were obtained.

## Results

### Sex ratio and pair composition

A total of 4587 leaf beetles were collected over the course of three years (Table 1). Overall, males (N_M_ = 2745) outnumbered females (N_F_ = 1842), although the sex ratio was not constant, being 1:0.45 in 2020 and 1:0.57 in 2022 (in 2021 we only sampled paired individuals, so the ratio was not calculated). We collected very few pregnant viviparous females (N = 15) (S3 and S4 Figs).

**Table 1:** Dataset composition. The number of adult leaf beetles sampled in the three years of the study (recorded as single or paired individuals in the 2021 and 2022 samplings, but unclassified in 2020).

Year	Category of individuals	Males	Females	Total
**2020**	*unclassified*	734	333	1067
**2021**	*heterosexual pairs*	849	849	1698
	*homosexual pairs*	6	-	6
**2022**	*singles*	637	217	854
	*heterosexual pairs*	437	437	874
	*homosexual pairs*	82	6	88
	*total*	2745	1842	4587

Most of the pairs collected in 2021 and 2022 were heterosexual, but a small minority comprised same-sex individuals (47 out of 1333, 3.52%), mostly male-male pairs. Male homosexual pairs were particularly numerous in 2022 (41 out of 481 pairs, 8.52%).

### Color polymorphism and morph frequencies

Once the color of each beetle had been acquired by the colorimeter, it was plotted within the CIE L*a*b* color space to define the relative position of all individuals.

**Fig 1:**
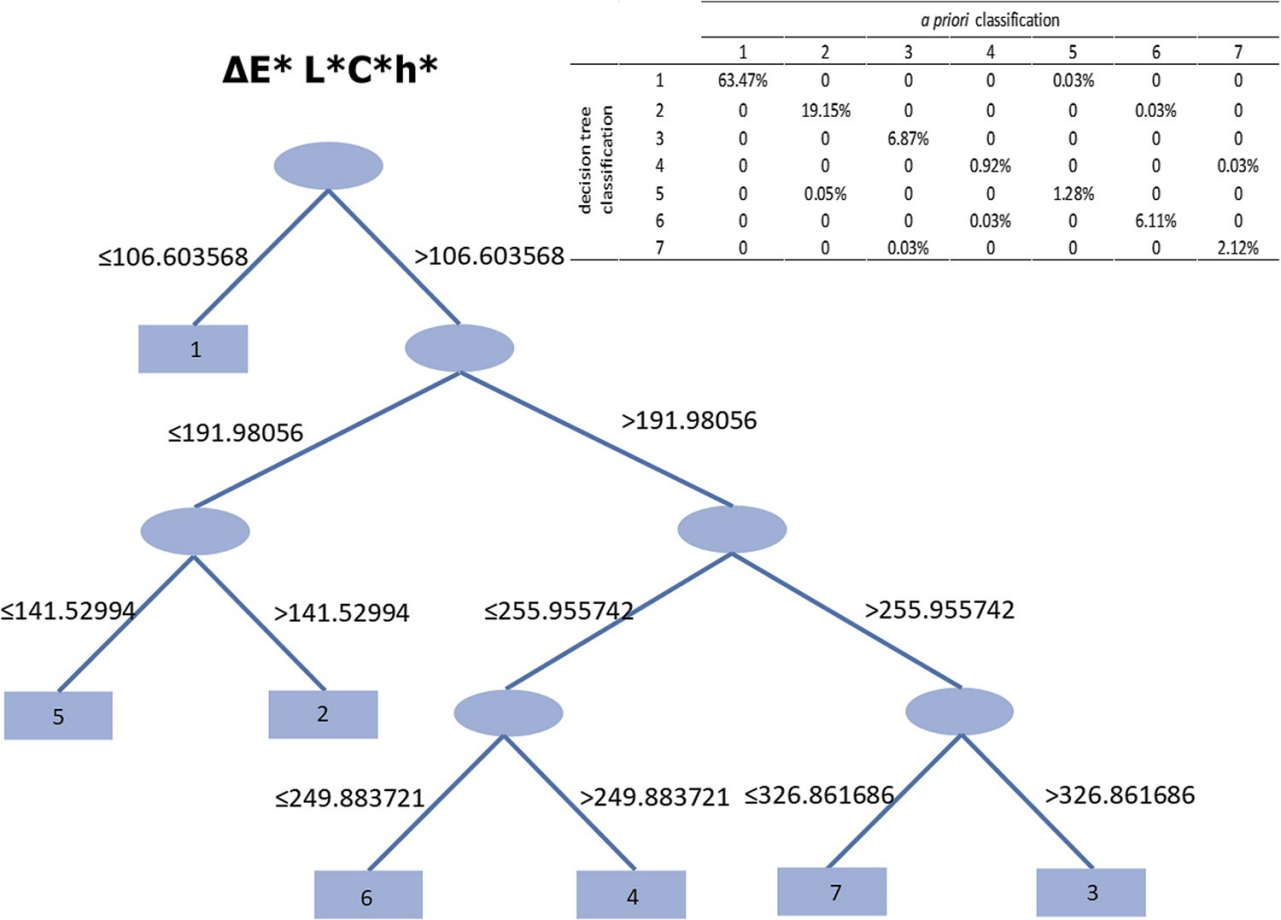
Decision tree. The DT was obtained using the J48 algorithm, considering the ΔE* L*C*h* color values to define *O*. *gloriosa* color morphs. Ovals represent the intermediate nodes; rectangles are the terminal “leaves” and the numbers within indicate the number of the morph identified. The percentages of correct classifications for the seven groups are shown in the confusion matrix (top right). The numbers above the connecting lines of the decision tree indicate the threshold values for each dichotomy. Kappa statistic = 0.9974; mean absolute error = 0.0004; root mean squared error = 0.0201; relative absolute error = 0.2565%; root relative squared error = 7.1649%.

At first glance, most of the individuals collected in the field generally appeared to be either green or blue. However, objective color assessment revealed a marked color polymorphism in the population. The J48 decision tree produced a classification comprising seven color morphs (four shared a green base color and three a blue base color), hereafter identified by the numbers 1–7. Analysis revealed the decision tree process to classify 99.86% of instances correctly, whereas 0.14% had been incorrectly classified (Fig 1). The accuracy of the classification according to class (i.e., color morph) confirmed proposed groups being all the weighted average measures significant (S1 Table).

Considering all the individuals collected in the three years, morph 1 (green-golden shade) was by far the most abundant (63.50%). The next most abundant was morph 2 (blue, 19.18%), followed by morphs 3 and 6, which showed similar abundances (6.87 and 6.14%, respectively). The least abundant were morphs 7 (2.15%), 5 (1.33%), and 4 (0.95%) (Fig 1, see confusion matrix).

**Fig 2:**
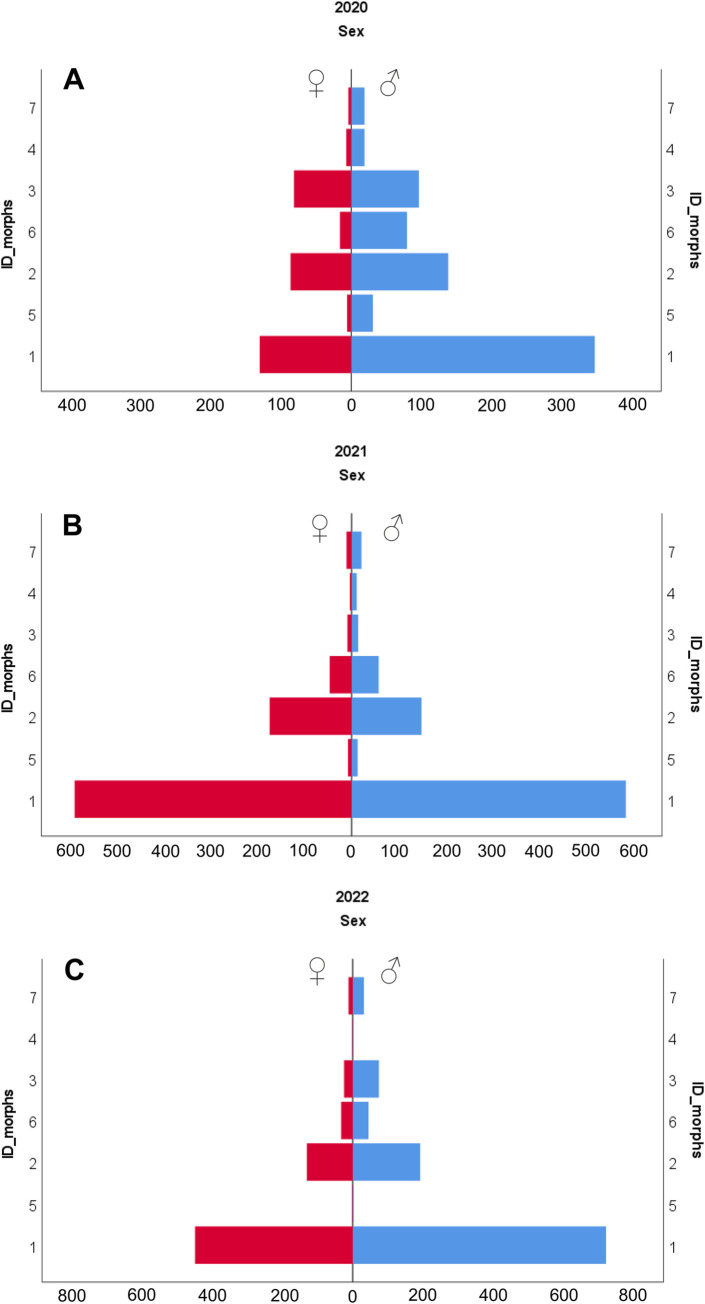
Abundances of the seven morphs for the years 2020, 2021, and 2022. The abscissa indicates the number of individuals of each sex: females on the left (red bars) vs males on right (blue bars). The number of morphs (1–7) are indicated on the vertical axis. Note, the sex ratio in 2021 was almost 1:1 (excluding same-sex couples) because only mating individuals were sampled that year.

The frequencies for the seven morphs were significantly different between the two sexes in the beetles sampled in 2020 (chi-square = 44.1, P = 0.0001), but not in 2021 (chi-square = 11.9, P = 0.064) or 2022 (chi-square = 4.27, P = 0.692). Instead, although morphs 1 and 2 (golden-green and blue, respectively) were the most abundant in all three years, relative frequencies of morphs were significantly different. This result was obtained both when only pairs of beetles were considered (i.e. 2021 vs 2022; males: chi square = 27.9, P<0.0001; females chi square = 12,7, P = 0.0448) and when all beetles were considered independent of being found as a pair or single individual (i.e. 2020 vs 2022; males: chi-square = 133, P<0.0001; females: chi-square = 150, P<0.0001) (Fig 2).

### Mating patterns

#### JMating

The significant total Ipsi value (P < 0.05 for all the bootstraps replications) confirmed that the effects of sexual isolation impacted mating patterns in the pairs collected in 2021. The results of G tests, which evaluated the effects of sexual selection and sexual isolation, separately and together (with significance values obtained using a chi square distribution), were as follows: GI = 58.68* (sexual isolation, df_I = 36), GS = 0 (sexual selection, df_S = 48), and GT = 58.65*** (total, df_T = 12). The 5x5, 3x3 and 7x7 pairs showed the highest PSI values among homotypic pairs, suggesting a high propensity for positive assortative mating (24.49, 13.48 and 8.09, respectively). Analogously, the 1x5 (male x female) heterotypic pair showed a very low value (0.18), suggesting they avoid each other, supporting the trend for positive assortative mating. Vice versa, heterotypic pairs 3x4, 5x4 and 7x5 (male x female in all cases) showed the highest values among all heterotypic pairs, suggesting negative assortative mating (Table 2). Apart from some instances of 0 values (because of the absence of the pair type in the field), all the remaining pairs had values slightly above or below 1, suggesting that the partners mated at random or in a weakly assortative manner (Table 2).

**Table 2:** PSI matrix. The indices are PSI values, where >1 indicates that the pair was observed more frequently than would be expected with random mating (suggesting positive assortative mating in homotypic pairs and negative assortative mating in heterotypic pairs) and <1 indicates it was observed less than expected than would be expected with random mating (suggesting a tendency toward positive assortative mating in heterotypic pairs and toward negative assortative mating in homotypic ones). The value 0 indicates that the pair was not observed in the field. Homotypic pairs are highlighted in gray along the diagonal.

	1	2	3	4	5	6	7
**1**	1.039	0.969	0.715	0.777	0.550	0.936	0.885
**2**	0.935	1.133	1.723	0.877	0.742	1.644	1.148
**3**	0.647	0.000	13.476	8.576	7.256	1.624	0.000
**4**	1.165	1.140	0.000	0.000	0.000	0.000	0.000
**5**	0.182	1.424	0.000	9.648	24.490	1.830	0.000
**6**	0.991	1.091	0.000	1.642	1.389	0.934	0.860
**7**	0.728	0.570	0.000	0.000	6.531	1.464	8.086

#### QInfomating

The significant values of deviation from random (Jpti = 0.112***) and assortative mating (Jpsi = 0.112***) led us to check the models of mutual mating propensity for the best fit, although sexual selection gave a non-significant value for both sexes independent of whether they were considered separately or together. The latter result was expected since the dataset only included the frequencies of the 2021 pairs (= mating individuals from the wild). The C-7P model (delta = 0.00, weight = 0.55, average propensity = 1.07) gave the AICc best-fit for PSI matrix frequencies from the 2021 dataset (NP_2021_ = 849) (Table 3), followed by SfemC-HP (delta = 1.89, weight = 0.21, average propensity = 1.05) and SmaleC-HP (delta = 1.92, weight = 0.21, average propensity = 1.05). Together, these three models accounted for 97% of the total weight, while the others were safely discarded, since their delta values were much higher, and their weight values were ≈ 0.00.

**Table 3:** The AICc output table. Female and male morphs are assigned to the rows and columns, respectively. The non-random mutual propensity diagonal relative to homotypic pairs is marked in gray. The numbers are the propensity to mate indexes, where >1: propensity to mate (-> positive assortative mating); 1: absence of propensity to mate (-> random mating); <1 mating avoidance (-> negative assortative mating). The C-7P model defines all the pairs as random (mate index = 1) except for the homotypic ones.

		♂						
		1	2	3	4	5	6	7
**♀**	**1**	1.11	1.00	1.00	1.00	1.00	1.00	1.00
	**2**	1.00	1.21	1.00	1.00	1.00	1.00	1.00
	**3**	1.00	1.00	14.41	1.00	1.00	1.00	1.00
	**4**	1.00	1.00	1.00	0.00	1.00	1.00	1.00
	**5**	1.00	1.00	1.00	1.00	26.19	1.00	1.00
	**6**	1.00	1.00	1.00	1.00	1.00	1.00	1.00
	**7**	1.00	1.00	1.00	1.00	1.00	1.00	8.65

The C-7P model represents not-random mating, meaning that morphs were inclined to select individuals of the same morph, although propensity values for each morph pair were different [39]. In this model, the color morphs 5, 3, and 7 showed high propensity for positive assortative mating (5 x 5 = 26.19; 3 x 3 = 14.41; 7 x 7 = 8.65). Morph 6 (6 x 6 = 1.00) and morphs 1 and 2 (1 x 1 = 1.11; 2 x 2 = 1.21) were considered as indicative of random mating or, at most, of a negligible tendency towards positive assortative mating (Table 3).

#### Montecarlo simulations

The results of the Montecarlo simulation carried on the 2022 data showed that, in most of the tested cases (44 out of 49), the observed mating frequencies lay within the percentile intervals (Table 4). Notably, we detected just a single homotypic morph pair (namely 1 x 1, the most abundant), for which the observed frequency was significantly higher than the simulated one, suggesting the occurrence of positive assortative mating. This type of mating was also indirectly supported by three heterotypic morph pairs (namely 1 x 7, 7 x 1, and 7 x 2, for males x females, respectively) in which the observed frequencies were significantly lower than the simulated ones (i.e., the morphs were avoiding each other). Finally, we detected a single heterotypic pair (namely 1 x 2) in which the observed frequencies were significantly higher than simulated ones, supporting negative assortative mating. (Table 4).

**Table 4:** Results of the Montecarlo analysis. The observed mating frequencies are reported in black if they are equal to the expected frequencies, in blue if significantly lower or in red if significantly higher.

		♀						
		1	2	3	4	5	6	7
♂	**1**	**50.1** **	**15.1** **	1.8	0.2	0	3.7	**0.5** **
	**2**	13.0	3.7	0.9	0	0	0.7	0
	**3**	3.7	0.9	0.2	0	0	0	0
	**4**	0	0.2	0	0	0	0	0
	**5**	0	0.2	0	0	0	0	0
	**6**	3.0	1.4	0	0	0	0	0.2
	**7**	**0.5** ***	**0** *	0	0	0	0	0

***p< = 0.001

** p<0.01

*p<0.05

### *Oreina gloriosa* as potential prey

The leaf beetles detected in the study area were all located on the leaves of masterwort, where they were highly visible and relatively immobile, but no predation attempts by birds or mammals were observed. Of the insectivorous birds spotted in the undergrowth, the wren *Troglodytes troglodytes* was the most common species. However, the wren, like the other species inhabiting the forest understory, usually perched on the arbors of bushes, and not on the flowers of herbaceous plants like masterwort.

It should be noted that individuals of *Oreina* were observed to perform thanatosis, letting themselves fall from the leaves inadvertently moved by the samplers (CP and ARL). About 5% of individuals managed to escape collection in the first days of sampling thanks to this anti-predator mechanism (we subsequently placed a net under the sampled leaf to catch any falling individuals).

## Discussion

The results of this study derive from intensive field sampling, with about 4600 individuals of *O*. *gloriosa* being collected from the same site over the course of three years. As far as we know, no other study on chrysomelid polymorphism has analyzed such a large sample of individuals.

### Sex ratio and pair composition

In the alpine population studied, the sex ratio for adult individuals collected in the field was biased in favor of males. Twice as many males were detected than females in both 2020 and 2022. This ratio is not in line with the sex ratio at birth, which is expected to be 1:1. Parity of the sexes at birth has been confirmed in breeding females of *Altica lythri* Aubé, 1843 [65]. However, it is plausible that the ratio in sexually mature insect populations may change over time and in relation to space, producing the so-called “tertiary sex ratio”, a reflection of the “*here and now”* situation. In *Diabrotica virgifera* LeConte, 1868 the sex ratio of adults was found to vary significantly over time and according to the sampling method used [66]. Interestingly, a study of natural populations of the leaf beetles *Altica brevicollis* Foudras, 1860 and *Gonioctena quinquepunctata* (Fabricius, 1787) showed that the sex ratio can change dramatically over time, primarily due to the disappearance of females after oviposition [67]. *Oreina gloriosa* is a viviparous species [68] and females about to give birth are easily identifiable by their enlarged abdomens. We detected very few of these females, which suggests that they hide when ready to give birth. The skewness of the sex ratio in favor of males can also explain why we sampled a certain number of male homosexual couples (especially in 2022), while female ones were virtually absent. These male-male couples were indistinguishable, in terms of behavior, from the heterosexual ones. Pairs of males could represent cases of scramble competition, even though the two individuals maintained the reciprocal position (one above the other) and did not give the impression of being engaged in interactions referable to scramble competition [69]. Alternatively, homosexual pairs may be interpreted as mating errors. For example, in high density species of mollusks females adaptively erase the sexual information present in the mucus to avoid (or reduce) mating; therefore, males fail to identify females and form homosexual pairs [70]. Populations of *O*. *gloriosa* may also reach high densities, such as when several individuals crowd on the same leaf. In these cases, females might provide false indications to males via pheromones, inducing them to mate erroneously with other males. Both hypotheses will need to be investigated in future studies.

### Color polymorphism and morph frequencies

The instrumental color analysis (using the colorimeter to represent colours in the CIE L*a*b* color space followed by classification by decision tree) led to the identification of seven color morphs, which correctly classified almost all the individuals collected (about 99.9%). It is worth noting that previous studies on chrysomelid polymorphism identified color morphs by sight alone. Most focused on the common morphs and considered the basal colors only [12, 14, 15], although one study did attempt to identify all morphs using the GIA color-grading scale [39]. In the present study, when considering the specimens collected across all three years, the overall population consisted of two common morphs (morphs 1 and 2, the former being the most abundant) plus five rarer morphs (morphs 3–7). However, it needs to be mentioned that the morph frequencies changed significantly over time. We collected all individuals present on the leaves of masterwort from the patch of forest hosting this insect, although their collection was split across four sampling days, one for each of the four areas which the patch was equally divided up into. Apart from a few exceptions, we failed to collect pregnant viviparous females, suggesting that many of them were hidden at that time to reproduce. Therefore, assuming an approximately constant birth rate, we believe that our collection procedure did not significantly influence either population dynamics or frequencies of morphs.

Color morph frequencies may also change among populations, controlled by demographic and climatic determinants. In *C*. *lapponica* the inter-population variation (light and dark morphs) depended on the type of the population and the stage of density change, with both color polymorphism and the frequency of dark morphs decreasing in post-outbreak density declining populations [19]. When looking at the inter-population differences in the frequencies of five color morphs of *C*. *lapponica*, characterized by different degrees of melanism, it was found that the highest level of polymorphism occurred at high latitudes and altitudes, presumably due to the different climatic requirements of coexisting color morphs [20]. However, these inter-population studies often assume that the frequencies of morphs are stable over time in the specific population studied. Our results provide evidence that the frequencies of morphs may change within a population over time, even on a year-to-year basis. Therefore, we suggest that caution be taken when drawing conclusions about differences in morph frequencies between spatially distinct populations, with emphasis being placed on only the most significant differences.

### Mating patterns

As explained in the introduction, our experimental design does not consider the possibility of properly estimating mating in the absence of SCE because we assumed it to be negligible. However, after three years of samplings, we realized that individuals appeared to move around very little, often remaining on the same leaves for a prolonged period of time. The fact that these insects performed thanatosis instead of flying away when we disturbed them during sampling may indeed confirm a low propensity for this insect to fly. To evaluate their movements, *ad hoc* studies should be planned and carried out, in which many individuals are marked and/or radio-tracked. As for evaluating the spatial distributions of the seven morphs, this is not possible as they cannot be differentiated in the field by sight. Therefore, all things considered, we cannot exclude *a posteriori* that the SCE may have influenced the results.

We analyzed 2021 and 2022 datasets using three distinct approaches to safeguard against missing findings due to the use of a single methodology.

JMating suggested positive assortative mating for the homotypic pairs of the morphs 3, 5, and 7 and for one heterotypic pair, and negative assortative mating for three heterotypic pairs. Most of the remaining pairs had PSI values weakly above or below 1, suggesting that the partners mated at random or in a weakly assortative manner. The best AICc model (C-7P) obtained with QInfomating confirmed that the same three morphs quoted above (3, 5 and 7) mated preferentially with individuals of the same color, whereas it indicated three other morphs to mate randomly or semi-randomly. The Montecarlo simulations indicated positive assortative mating for the homotypic pairs of morph 1 (which was, notably, the most abundant) and for three heterotypic pairs. The analysis also suggested a case of negative assortative mating.

Hence, while the results obtained with JMating and QInfomating (2021 sample) and Montecarlo simulations (2022 sample) are not the same, they agree in depicting a mating trend in which random mating may coexist with some instances of positive and negative assortative mating. This could partly explain the pre-eminence of one morph (which would be favored because of positive selection due to the presence of positive assortative mating) and, at the same time, the persistence of the other morphs (which would be maintained because of negative selection due to cases of negative assortative mating).

These results suggest that frequency-dependent selection caused by assortative mating may contribute to maintaining color polymorphisms in these chemically defended leaf beetles, in keeping with other experimental and theoretical papers [34, 71]. However, since we cannot exclude the presence of SCE, further studies employing designs and analyses aimed at rejecting this possibility must be conducted in the future.

In general, positive assortative mating may evolve to avoid species confusion, i.e. as a defense against species interbreeding or at least against wasting time trying to mate with a different species [72]. In theory, this may even be what is happening in *O*. *gloriosa*, given that there are at least another five species of *Oreina* with similar colors residing in the Alps [12, 14, 16], although these species are not cryptic as they exhibit different morphological (body size, male and female genitalia) and physiological traits (different types of chemical defenses), suggesting that the degree of interspecific confusion, if any, may be low.

That said, just because individuals of *O*. *gloriosa* tend to mate with individuals of the same or different color does not prove incontrovertibly that color is the discriminating factor in mate choice. A particular difficulty in color perception could depend on UV reflectance. Many beetles reflect UV [73] and it is likely that leaf beetles do too. That said, the visual abilities of these insects do consent to the possibility that mate discrimination is based on color [74] and it is in line with some observations suggesting that color may be used in mate choice [33]. Positive assortative mating in leaf beetles has been assessed in relation to the cuticular hydrocarbon profile [32], beetle size [30] and parasite load [31]. However, no data is available on whether the different color morphs are associated with different hydrocarbon profiles, body sizes or different parasitic loads, and, therefore, whether mate selection is influenced by these characteristics and not by color. Finally, we cannot exclude the possibility that mate selection is based on unknown characteristics associated with the different color morphs.

### On predation

The *O*. *gloriosa* leaf beetles sampled were highly visible and noted as being relatively immobile. As such, they would be expected to be at risk of predation, but no attempts by birds or mammals were witnessed, even when the observer was hidden. Small insectivorous passerines may capture these insects by hovering at leaf height or by perching on the host plant. However, no such bird species in Europe are capable of hovering (like hummingbirds), and the birds that would be able to perch on masterwort (such as the whinchat *Saxicola rubetra*) live in open rough pastures and grassland, and not in forest habitats (ARL, personal communication). The results of recent studies conducted on captive great tits, *Parus major* Linnaeus, 1758 preying on *Chrysomela lapponica* [13] and on blue tits *Cyanistes caeruleus* (Linnaeus, 1758) preying on *O*. *alpestris* Schummel, 1843 and *O*. *cacaliae* [16] suggest that these insectivorous birds can associate color with the chemical defenses of leaf beetles. These two tit species should be considered *potential* predators since they have never been observed to prey on leaf beetles in the wild. Both species are arboreous insect predators, especially the blue tit. We can assume, therefore, that although the predation of great tits on *Chrysomela lapponica*, which feeds on willow, is plausible, that of great tits on *O*. *alpestris* and *O*. *cacaliae*, which feed respectively on Apiaceae and Asteraceae on the ground, is less likely. Another study, conducted in the wild on *O*. *gloriosa* leaf beetles tethered by means of fine plastic leashes, demonstrated the occurrence of frequency-dependent selection due to predation [11]. However, the authors did not identify the predator and affirmed that potential predators of the adults include many birds that hunt visually, such as the robin *Erithacus rubecula* (Linnaeus, 1758), wren *Troglodytes troglodytes* (Linnaeus, 1758), and dunnock *Prunella modularis* (Linnaeus, 1758) [11]. These three bird species feed among shrubs and bushes, but their ability to perch on slender umbellifer plants, like masterwort, has yet to be ascertained.

In this study, leaf beetles would often escape collection by hand from the host plant by letting themselves fall off into the undergrowth. Thus, the predator defense mechanisms in place in these animals appear to be very effective and involve thanatosis–the classic anti-predator behavior in many beetle species–in addition to chemical defenses. All considered, given the potential role of predation in explaining the evolution and ecology of the color-based defense mechanisms of Chrysomelidae, we suggest that future research be directed toward identifying the predators of these chemically defended insects.

## Supporting information

S1 Fig Selected position for the color acquisition. To obtain comparable reflectance values the measurement caliper was placed always on the same portion of the elytral surface.
(PDF)

S2 Fig Classification of the color morphs in the *O*. *gloriosa* population. The *a priori* classification of the seven color morphs was made using together the values of ΔE*L*a*b* (on the x-axis) and those of ΔE*L*C*h* (on the y-axis): the two datasets of the distances (ΔE*L*a*b* and ΔE*L*C*h*) were plotted to show the relative position of all the individuals within the population. The relative position of the individuals was compared against their color features to define the number of morphs and the threshold values. The best results were obtained considering a 7-morph classification system.
(PDF)

S3 Fig The viviparity in the alpine leaf beetle *O*. *gloriosa*. **A.** Tomogram of the abdomen of a non-pregnant female. **B.** Tomogram of the abdomen of a pregnant female, showing the preimaginal instars. A few pregnant females (N = 15) were dissected. Embryos harbored in the dilated abdomens ranged from 40 to 100 (60 on average). They were larger and more developed near the pygidium while they were smaller and less developed towards the interior of the abdomen. Some females whose abdomens were not expanded were also examined, and an average of 25 embryos (smaller than those of the heavily pregnant females) were found in these individuals. It therefore follows that the total number of offspring produced per female was probably underestimated because the collection and preservation in alcohol interrupted the maturation of other offspring. For more information about the tomograph settings please refer to the paper of Kerman *et al*. (2018 https://doi.10.3390/insects9030108).
(PDF)

S4 Fig The viviparous female of *O*. *gloriosa*. **A.** The dissected abdomen of a pregnant female. **B.** The position of the preimaginal instars within each tubular ovariole. **C-E.** Preimaginal instars at different level of development. **D.** The abdomen with the tubular ovarioles exposed. **F**. Details of the head showing the developing mouthparts.
(PDF)

S1 Table Accuracy of the classification obtained by the J48 decision tree. TP rate = true positive rate; FP rate = false positive rate; precision = percentage of predictions made by the model that are correct; Recall = percentage of relevant data points that were correctly identified by the model; F-measure = measure of the test’s accuracy; MCC = measure of the difference between the predicted values and actual values; ROC area = measure of the model’s efficiency; PRC area = measure of the relationship between precision and recall.
(PDF)

S1 Graphical abstract (TIF)
