# Correction: Color polymorphism and mating trends in a population of the alpine leaf beetle *Oreina gloriosa*

**DOI:** 10.1371/journal.pone.0324643

**Published:** 2025-05-22

**Authors:** The PLOS One Staff

The Funding statement for this paper is incorrect. The correct Funding statement is as follows: National Recovery and Resilience Plan (NRRP) Spoke 3 Mission 4 “Education and Research”, Component 2 “From Research to Business” (MUR-M4C2) Strengthening research structures and creating R&D “national champions (MUR CN00000033, CUP D13C22001350001).

The publisher apologizes for the error.

## References

[1] Roggero A, Alù D, Laini A, Rolando A, Palestrini C. Color polymorphism and mating trends in a population of the alpine leaf beetle Oreina gloriosa. PLoS One. 2024;19(3):e0298330. doi: 10.1371/journal.pone.0298330 38530852 PMC10965098

