# Supplementary material for: Color polymorphism and mating trends in a population of the alpine leaf beetle Oreina gloriosa
Source: PLoS One. 2024 Mar 26;19(3):e0298330. doi: 10.1371/journal.pone.0298330 (PMC10965098; doi:10.1371/journal.pone.0298330)
Supplement: S1 Fig — To obtain comparable reflectance values the measurement caliper was placed always on the same portion of the elytral surface. (PDF) [file pone.0298330.s001.pdf]

Supporting Information

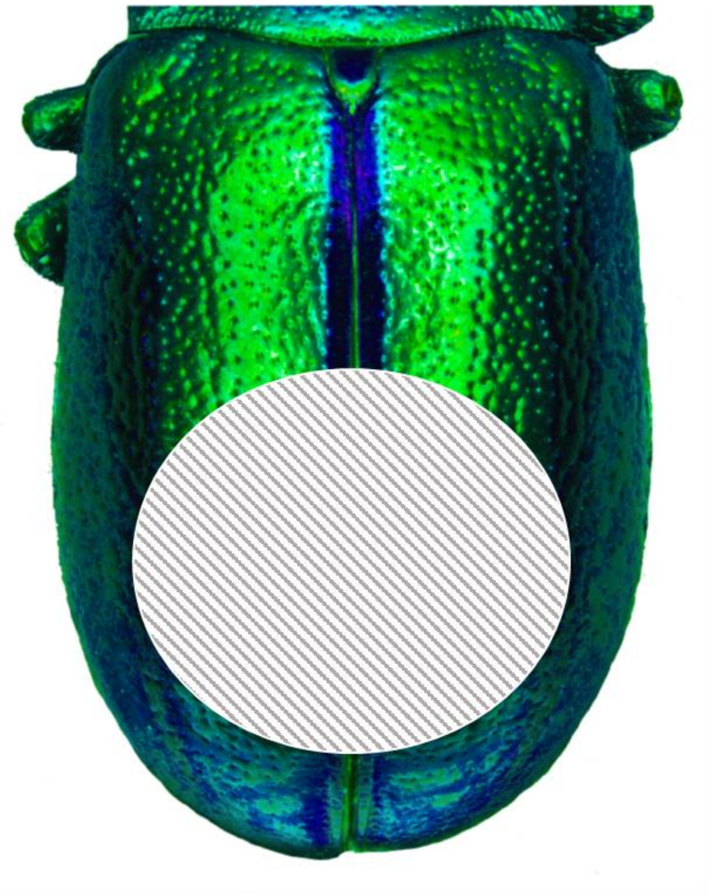

Figure S1. Selected position for the color acquisition. To obtain comparable reflectance values the measurement caliper was placed always on the same portion of the elytral curved surface.
