# Supplementary material for: Color polymorphism and mating trends in a population of the alpine leaf beetle Oreina gloriosa
Source: PLoS One. 2024 Mar 26;19(3):e0298330. doi: 10.1371/journal.pone.0298330 (PMC10965098; doi:10.1371/journal.pone.0298330)
Supplement: S2 Fig — The a priori classification of the seven color morphs was made using together the values of ΔE*L*a*b* (on the x-axis) and those of ΔE*L*C*h* (on the y-axis): the two datasets of the distances (ΔE*L*a*b* and ΔE*L*C*h*) were plotted to show the relative position of all the individuals within the population. The relative position of the individuals was compared against their color features to define the number of morphs and the threshold values. The best results were obtained considering a 7-morph classification system. (PDF) [file pone.0298330.s002.pdf]

Supporting Information

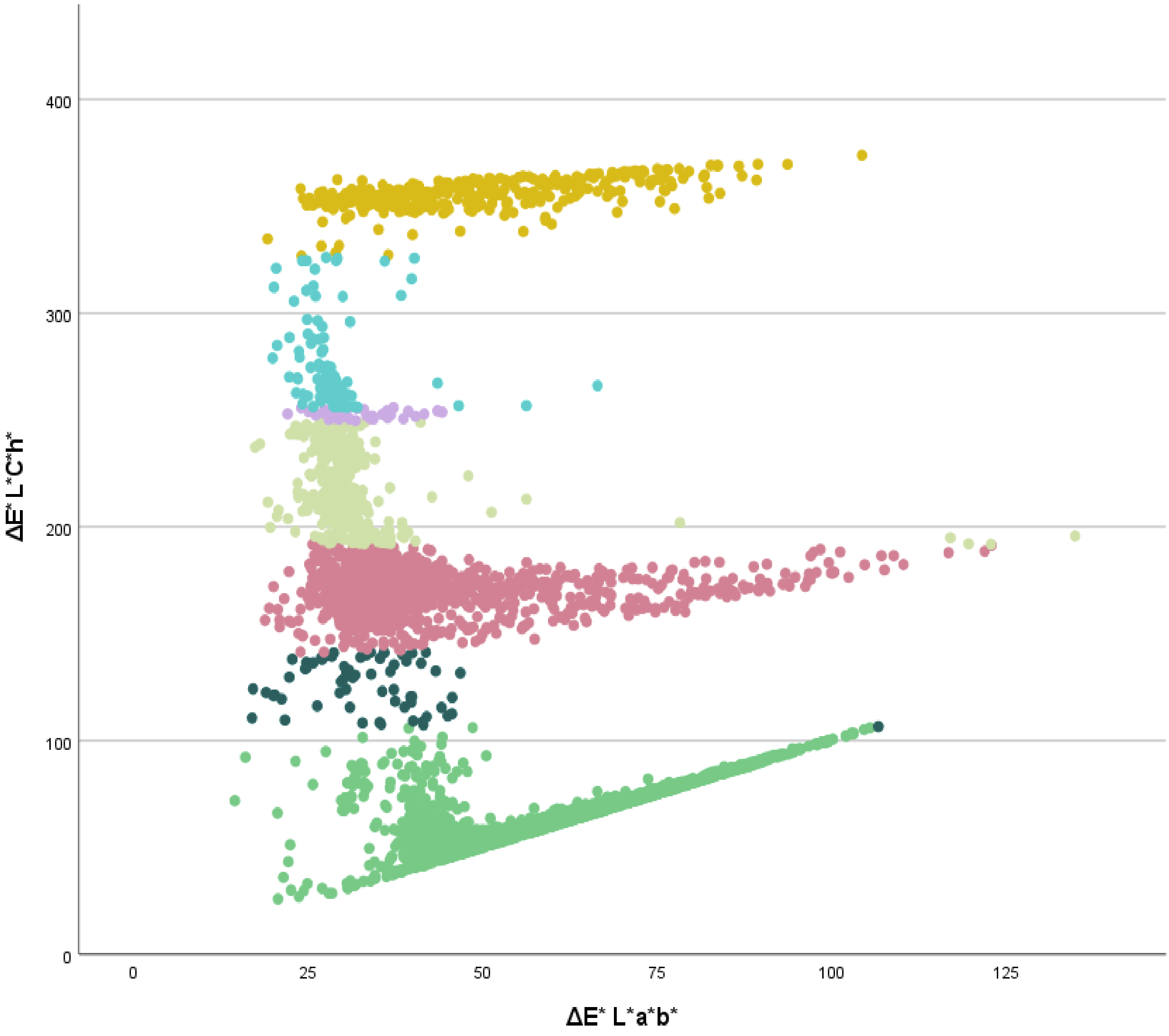

Figure S2. Classification of the color morphs in the *O. gloriosa* population. The *a priori* classification of the seven color morphs was made using together the values of ΔE*L*a*b* (on the x-axis) and those of ΔE*L*C*h* (on the y-axis): the two datasets of the distances (ΔE*L*a*b* and ΔE*L*C*h*) were plotted to show the relative position of all the individuals within the population. The relative position of the individuals was compared against their color features to define the number of morphs and the threshold values. The best results were obtained considering a 7-morph classification system.
