# Supplementary material for: Color polymorphism and mating trends in a population of the alpine leaf beetle Oreina gloriosa
Source: PLoS One. 2024 Mar 26;19(3):e0298330. doi: 10.1371/journal.pone.0298330 (PMC10965098; doi:10.1371/journal.pone.0298330)
Supplement: S3 Fig — A. Tomogram of the abdomen of a non-pregnant female. B. Tomogram of the abdomen of a pregnant female, showing the preimaginal instars. A few pregnant females (N = 15) were dissected. Embryos harbored in the dilated abdomens ranged from 40 to 100 (60 on average). They were larger and more developed near the pygidium while they were smaller and less developed towards the interior of the abdomen. Some females whose abdomens were not expanded were also examined, and an average of 25 embryos (smaller than those of the heavily pregnant females) were found in these individuals. It therefore follows that the total number of offspring produced per female was probably underestimated because the collection and preservation in alcohol interrupted the maturation of other offspring. For more information about the tomograph settings please refer to the paper of Kerman et al. (2018 https://doi.10.3390/insects9030108). (PDF) [file pone.0298330.s003.pdf]

Supporting Information

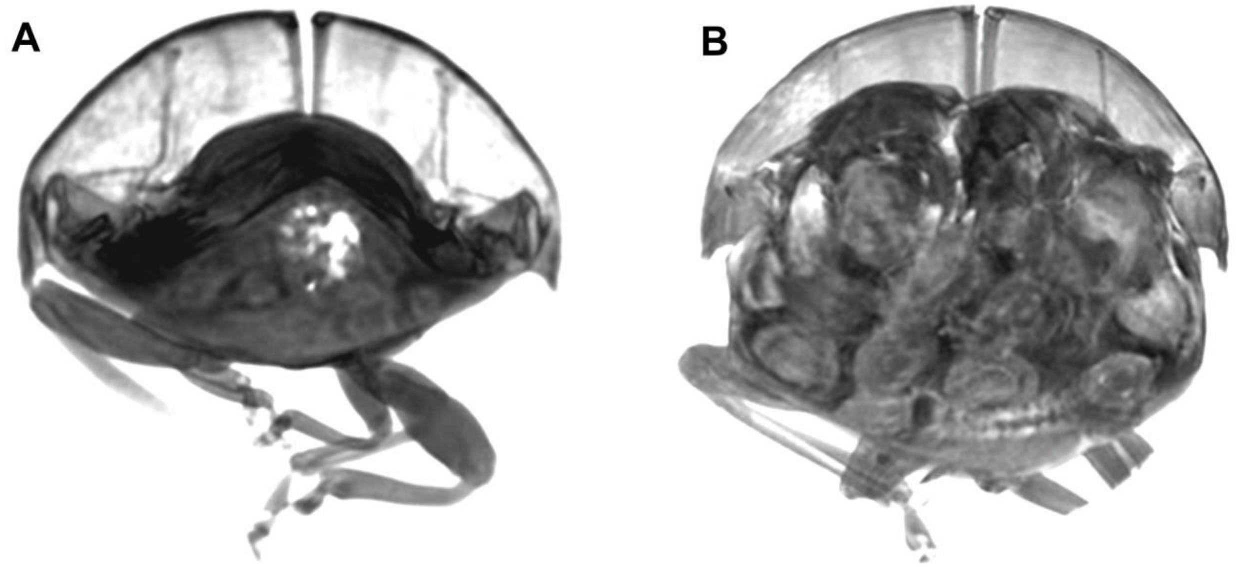

Figure S3. The viviparity in the alpine leaf beetle *O. gloriosa*. A. Tomogram image of the abdomen of a non-pregnant female. **B.** Tomogram image of the abdomen of a pregnant female, showing the preimaginal instars. A few pregnant females (N= 15) were dissected. Embryos harbored in the dilated abdomens ranged from 40 to 100 (60 on average). They were larger and more developed near the pygidium while they were smaller and less developed towards the interior of the abdomen. Some females whose abdomens were not expanded were also examined, and an average of 25 embryos (smaller than those of the heavily pregnant females) were found in these individuals. It therefore follows that the total number of offspring produced per female was probably underestimated because the collection and preservation in alcohol interrupted the maturation of other offspring. For more information about the tomograph settings please refer to the paper of Kerman *et al.* (2018 https://doi.10.3390/insects9030108)
