# Supplementary material for: Color polymorphism and mating trends in a population of the alpine leaf beetle Oreina gloriosa
Source: PLoS One. 2024 Mar 26;19(3):e0298330. doi: 10.1371/journal.pone.0298330 (PMC10965098; doi:10.1371/journal.pone.0298330)
Supplement: S4 Fig — A. The dissected abdomen of a pregnant female. B. The position of the preimaginal instars within each tubular ovariole. C-E. Preimaginal instars at different level of development. D. The abdomen with the tubular ovarioles exposed. F. Details of the head showing the developing mouthparts. (PDF) [file pone.0298330.s004.pdf]

Supporting Information

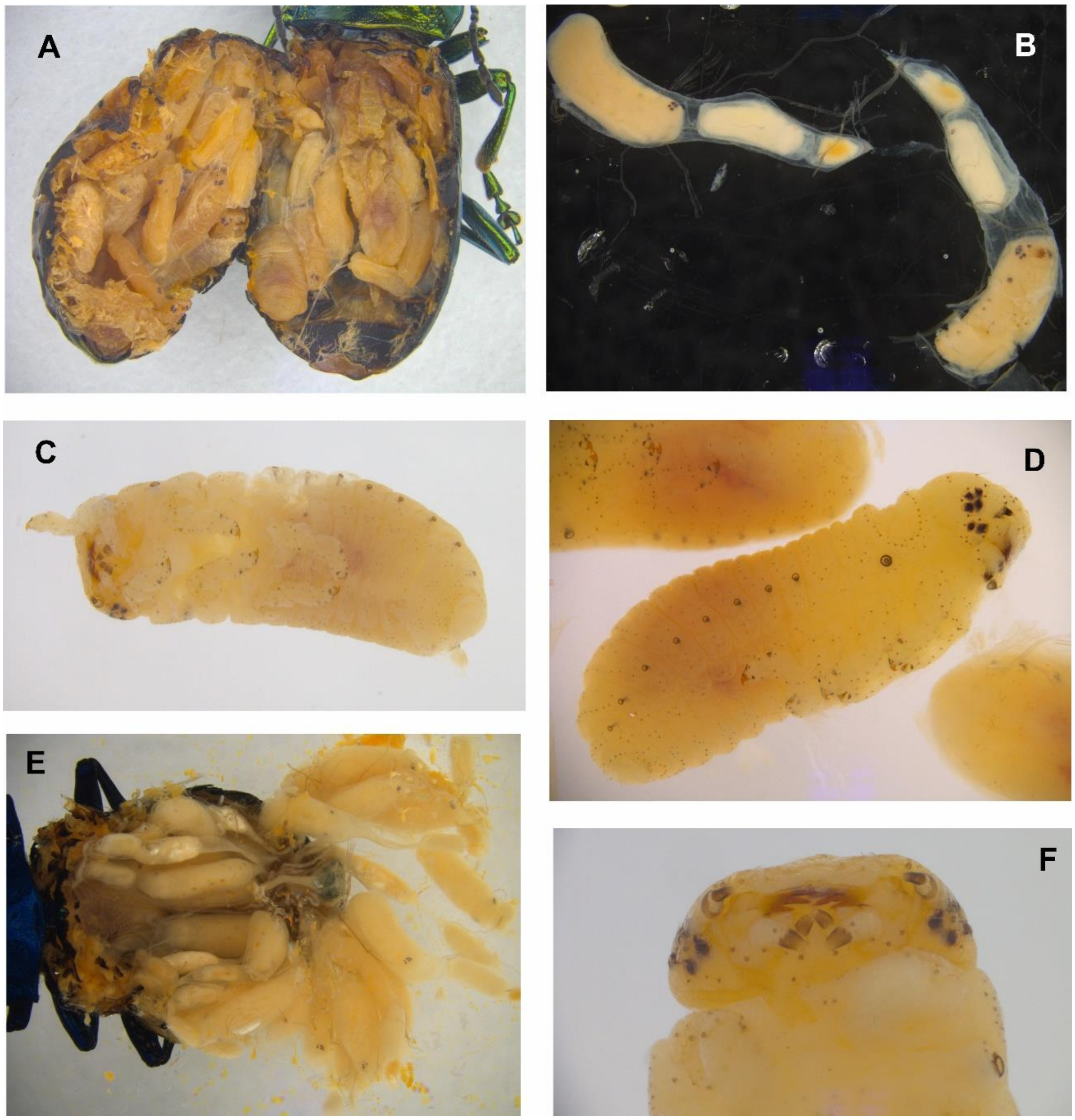

Figure S4. The viviparous female of *O. gloriosa*. A. The dissected abdomen of a pregnant female. **B.** The position of the preimaginal instars within each tubular ovariole. **C** and **D.** Preimaginal instars at different level of development. **E**. The abdomen with the tubular ovarioles exposed. **F.** Details of the head showing the developing mouthparts.
