# Supplementary material for: Color polymorphism and mating trends in a population of the alpine leaf beetle Oreina gloriosa
Source: PLoS One. 2024 Mar 26;19(3):e0298330. doi: 10.1371/journal.pone.0298330 (PMC10965098; doi:10.1371/journal.pone.0298330)
Supplement: S1 Table — TP rate = true positive rate; FP rate = false positive rate; precision = percentage of predictions made by the model that are correct; Recall = percentage of relevant data points that were correctly identified by the model; F-measure = measure of the test’s accuracy; MCC = measure of the difference between the predicted values and actual values; ROC area = measure of the model’s efficiency; PRC area = measure of the relationship between precision and recall. (PDF) [file pone.0298330.s005.pdf]

Supporting Information

Table S1. Accuracy of the classification obtained by the J48 decision tree. TP rate = true positive rate; FP rate = false positive rate; precision = percentage of predictions made by the model that are correct; Recall = percentage of relevant data points that were correctly identified by the model; F-measure = measure of the test's accuracy; MCC = measure of the difference between the predicted values and actual values; ROC area = measure of the model's efficiency; PRC area = measure of the relationship between precision and recall.

Class	TP Rate	FP Rate	Precision	Recall	F-Measure	MCC	ROC Area	PRC Area
**1**	1.000	0.000	1.000	1.000	1.000	1.000	1.000	1.000
**2**	0.999	0.001	0.998	0.999	0.998	0.998	0.999	0.997
**3**	1.000	0.000	0.997	1.000	0.999	0.998	1.000	0.997
**4**	0.978	0.000	0.978	0.978	0.978	0.978	0.989	0.957
**5**	0.969	0.000	0.984	0.969	0.977	0.976	0.985	0.954
**6**	0.997	0.000	0.997	0.997	0.997	0.996	0.998	0.994
**7**	0.991	0.000	0.991	0.991	0.991	0.99	0.995	0.981
**Weighted Avg.**	0.999	0.000	0.999	0.999	0.999	0.998	0.999	0.997
