# Supplementary figures and images for: Color polymorphism and mating trends in a population of the alpine leaf beetle Oreina gloriosa
Source: PLoS One. 2024 Mar 26;19(3):e0298330. doi: 10.1371/journal.pone.0298330 (PMC10965098; doi:10.1371/journal.pone.0298330)

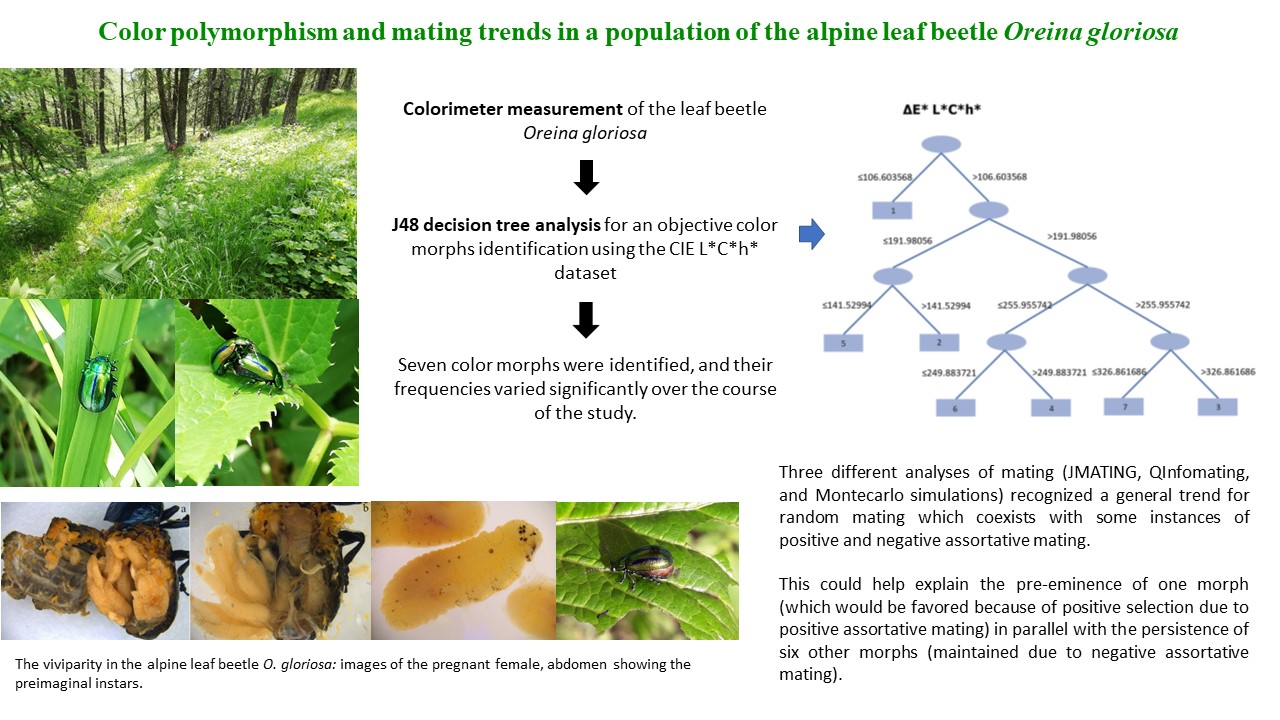

Supplement: S1 Graphical abstract — (TIF) [file pone.0298330.s006.tif]
